# Nanostructured Graphene: An Active Component in Optoelectronic Devices

**DOI:** 10.3390/nano8050328

**Published:** 2018-05-14

**Authors:** Chang-Hyun Kim

**Affiliations:** Department of Electronic Engineering, Gachon University, Seongnam 13120, Korea; chang-hyun.kim@gachon.ac.kr; Tel.: +82-31-750-8850

**Keywords:** nanostructured graphene, hybrid nanotechnology, chemical functionalization, optoelectronics, semiconductor devices

## Abstract

Nanostructured and chemically modified graphene-based nanomaterials possess intriguing properties for their incorporation as an active component in a wide spectrum of optoelectronic architectures. From a technological point of view, this aspect brings many new opportunities to the now well-known atomically thin carbon sheet, multiplying its application areas beyond transparent electrodes. This article gives an overview of fundamental concepts, theoretical backgrounds, design principles, technological implications, and recent advances in semiconductor devices that integrate nanostructured graphene materials into their active region. Starting from the unique electronic nature of graphene, a physical understanding of finite-size effects, non-idealities, and functionalizing mechanisms is established. This is followed by the conceptualization of hybridized films, addressing how the insertion of graphene can modulate or improve material properties. Importantly, it provides general guidelines for designing new materials and devices with specific characteristics. Next, a number of notable devices found in the literature are highlighted. It provides practical information on material preparation, device fabrication, and optimization for high-performance optoelectronics with a graphene hybrid channel. Finally, concluding remarks are made with the summary of the current status, scientific issues, and meaningful approaches to realizing next-generation technologies.

## 1. Introduction

As the technological needs of the modern society have become more diversified than ever, it might be desirable to create new electronic materials that can provide on-demand functions every time a new need arises. However, given the limited possibility of synthesizing or isolating totally new materials, hybridized use of known materials can be a smart yet realistic alternative, and this has indeed become an important trend in current materials science and electronics research. Notable examples are organic-inorganic hybrid thin-film devices [1,2,3] and mixed-dimensional van der Waals heterostructures [4,5,6,7], which are shown to be able to not only combine existing strengths of ingredients, but also generate synergistic effects based on interface interaction.

In this context, graphene-based nanohybrid materials can be considered as a versatile platform for next-generation optoelectronics. Since its first detailed characterization reported in 2004, graphene has revealed its extraordinary electronic, optical, thermal, and mechanical properties, which are particularly promising for use as a transparent and flexible electrode in conventional semiconductor devices [8,9,10,11]. More recently, new solution-based synthesis and functionalization techniques have allowed for the incorporation of graphene as a versatile channel material, the properties of which can be fine-tuned for targeted functionalities. These channel structures are often formed as hybrid nanocomposites between size-controlled, modified graphene and other semiconductors, and such hybrids have shown substantial performance improvements and/or tunability as compared to pristine materials. The emergence of several reviews dedicated to hybrids and composites underlines the growing interest in this concept as new opportunities for graphene [12,13,14].

However, the observed device behaviors tend to vary widely between architectures and often seem not to be sufficiently rationalized, which can be above all due to the complex nanoscale phases and interfaces that dominate effective macroscopic functionalities. Acknowledging this issue, the purpose of this review is to provide essential understanding on the governing structural and physicochemical properties of active hybrid materials incorporating nanostructured graphene and to link this understanding to reviewing and digesting state-of-the-art devices. Such a procedure is intended to fill the conceptual gap between proposed devices and observed performances, and it is expected to also provide practical guidelines for designing new hybrid materials and devices with tailored characteristics.

## 2. Basic Concepts

### 2.1. Pristine Graphene

**Figure 1:**
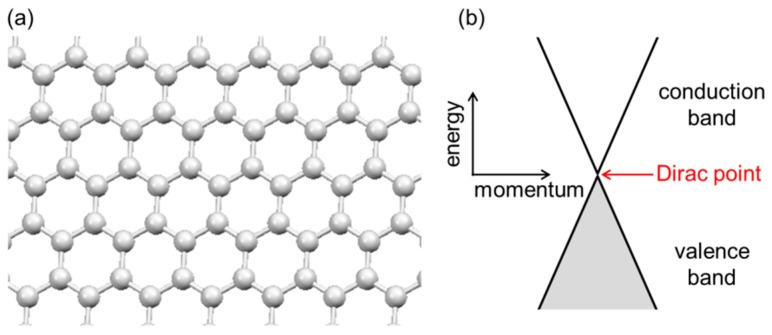
(**a**) Shape of a monolayer graphene sheet; (**b**) electronic band structure of pristine graphene.

Graphene is a two-dimensional honeycomb network of sp^2^-hybridized carbon atoms, each of which donates one *π* electron that is delocalized to carry electrical conductivity over the entire lattice structure (Figure 1a). Positional symmetry and periodicity in pristine and ‘ideal’ graphene (i.e., infinite size, perfect crystal, no impurities) gives rise to an unusual electronic band structure; as illustrated in Figure 1b, the material’s filled valence band and empty conduction band have a conical shape in the energy-momentum space (these bands are often called Dirac cones), and these two bands meet at the Dirac point. This energetic situation dictates the special property of graphene as a ‘zero-gap semiconductor’. While exhibiting metallic characteristics, it is unlike traditional metals that have an overlap between the two bands. Simultaneously, graphene differs from conventional semiconductors in that there is no band gap.

**Figure 2:**
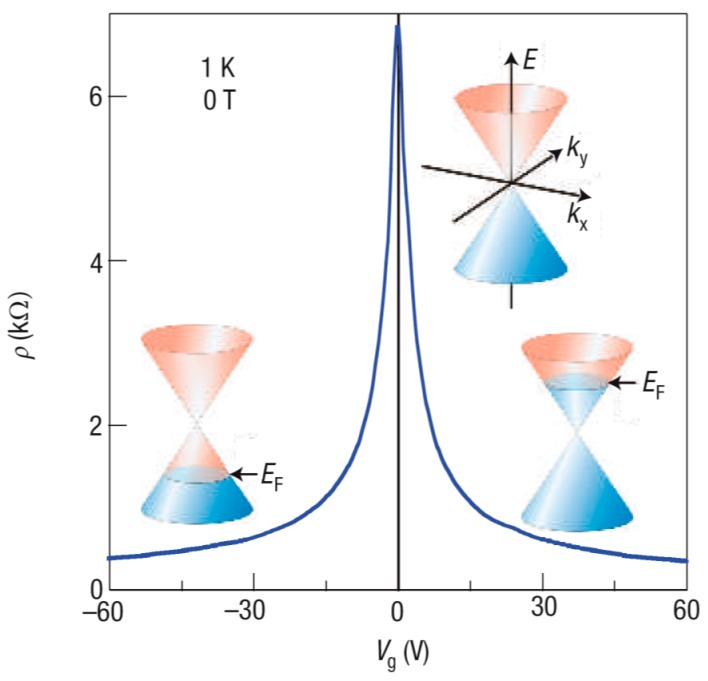
Characteristic field-effect behavior in single-layer graphene with its resistivity *ρ* decreasing by adding either holes (at negative *V_G_*) or electrons (at positive *V_G_*). *E_F_* is the Fermi level. Reproduced with permission from [17]. Nature Publishing Group, 2007.

In the absence of an electric field and near absolute zero temperature, the Fermi level is positioned near the Dirac point, and the electrical conductivity of graphene shows a minimum value due to a low free-carrier density. By applying an external gate electric field, the Fermi level can be moved toward and into the energy bands, and its departure from the Dirac point results in the increase in hole or electron density (depending on the direction of Fermi level movement). This explains symmetric V-shape ambipolar transfer curves (gate voltage *V_G_* versus drain current *I_D_*) or equivalent Λ-shape resistivity-*V_G_* plots of a graphene channel field-effect transistor (FET) (Figure 2) [15,16,17]. Such a simple device has been widely investigated to probe intrinsic physical properties of graphene, and it is believed that the low level of the on-off current ratio (generally less than 10 at room temperature) might be tolerable for certain applications such as logic circuits [18]. However, making graphene less metallic and more semiconducting with a high charge-carrier mobility and reduced off-state conduction has become a meaningful research motivation, because the realization of this goal can considerably enlarge the technological application window of graphene material [19].

### 2.2. Size Effects

Roughly speaking, finite-sized graphene is expected to have less metallic properties as compared to a large-area or quasi-infinite graphene sheet considered above. This is an interesting thing to keep in mind when creating hybrid material systems, especially composites, where graphene generally exists as small-size flakes embedded and distributed in a larger host matrix.

**Figure 3:**
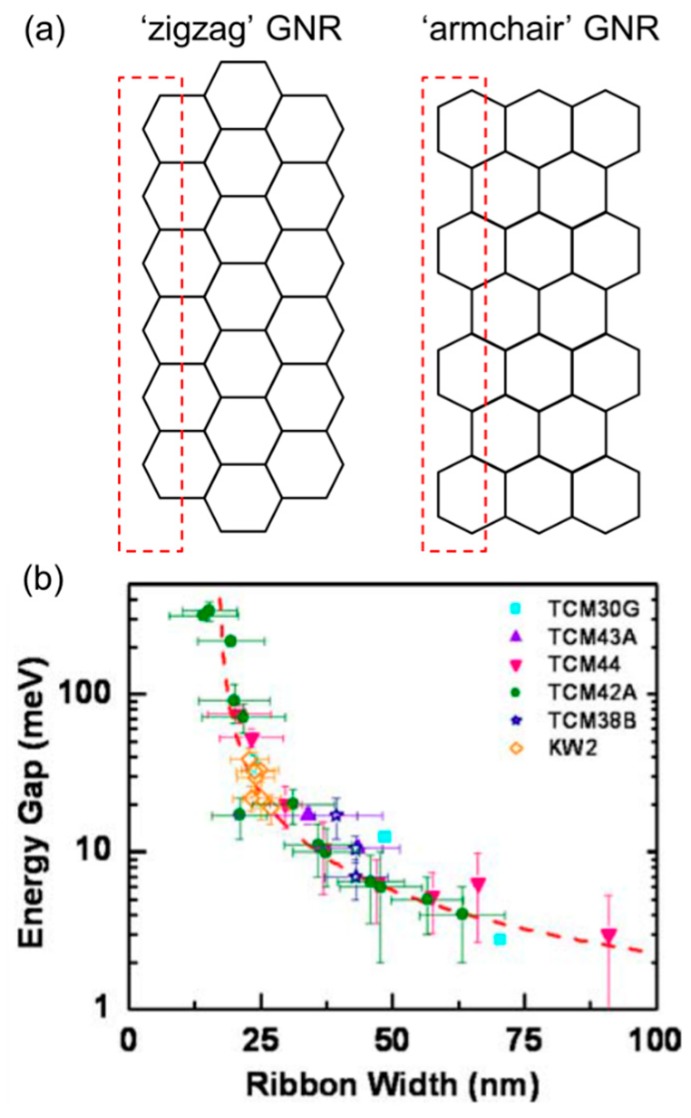
(**a**) Illustration of the two characteristic graphene nanoribbon (GNR) motifs, namely zigzag and armchair, as determined by the repeating edge pattern; (**b**) Experimentally measured energy gap as a function of GNR width. These data were extracted from several devices with different sizes and orientations. Reproduced with permission from [23]. Wiley-VCH, 2007.

The theory of graphene nanoribbons (or GNRs), which are long and narrow strips made of graphene, helps to understand this. GNRs were proposed as an attempt to make graphene more switchable in transistors. Computational studies have shown a possible energetic gap opening in certain geometries, with predictable changes [20,21,22]. Reducing the structural repetition in one of the two dimensions results in the limited splitting of atomic orbitals in that direction, and this confinement can eventually open the energy band gap. Detailed analyses have shown that the band structures of GNRs are strongly affected by the crystallographic orientation or edge pattern; zigzag GNRs are normally metallic, while armchair GNRs can be either metallic or semiconducting (Figure 3a). The confinement effect and resulting band alteration becomes stronger when the ribbon width shrinks. Han et al. fabricated a series of shape-controlled GNRs by e-beam lithography and oxygen plasma etch and proved that the energy gap is inversely proportional to the GNR width, with a sizeable gap of ~200 meV in the case of a ~15 nm-wide sample (Figure 3b) [23]. Other reports employing a GNR channel in FET architecture showed appreciable current on-off ratios of the order of 10^3^ or 10^4^ [24,25]; values that clearly evidence the gap opening and semiconducting behavior. Advanced preparation methods for GNRs are also gaining significant attention. For instance, ‘on-surface’ direct synthesis of GNRs has been recently proposed, with extremely high structural controllability and possibilities for accurate material characterization by scanning-tunneling spectroscopy (STS) [26,27].

With GNRs being a relatively well-studied and well-defined model system, there are many other possible reduced-size nanostructures that can be obtained from both bottom-up and top-down approaches [28]. The lessons learned from GNRs would allow for the prediction that the size seen along different axes, the direction(s) of confinement, and the edge roughness of any arbitrary-shape graphene-derived nanomaterials would be the major factors that would determine the electronic properties of these materials. Also, it is important to note that the extended tunability from physical structuring and the possibility for converting between metallic and semiconducting states is one of the elements that underlines the remarkable versatility of graphene-based optoelectronics.

Another example of structural confinement is graphene nanomesh, proposed by Bai et al. in 2010 [29]. It refers to a hierarchical material that is basically a graphene sheet with a high-density array of nanoscale holes. Nanomeshes seemed to be especially advantageous for FETs, as they could sustain an overall large in-plane conductivity (due to the long-range connectivity), while featuring a certain bandgap at the same time (due to the occasional breaks in coupling). Schmidt et al. very recently demonstrated a suspended 10-nm pitch graphene nanomesh [30], which is attractive for investigating intrinsic physical properties without substrate effect and for eventual double-side functionalization.

### 2.3. Surface Doping

So far, size and orientation control of graphene has been considered as a type of structural engineering without the incorporation of any foreign chemical species or impurities. Another necessary concept is the doping of graphene, which provides an additional and rich tunability just as in conventional semiconductors. Similar to molecular and polymeric semiconductors, however, the major doping mechanism in graphene is not substitutional (at the atomic level) but rather relies on the charge transfer between the two materials forming tight interfaces [31,32,33]. Therefore, good examples for investigating doping are a graphene sheet on which charge-donating or accepting species are deposited. These materials induce the ‘surface doping’ of graphene.

**Figure 4:**
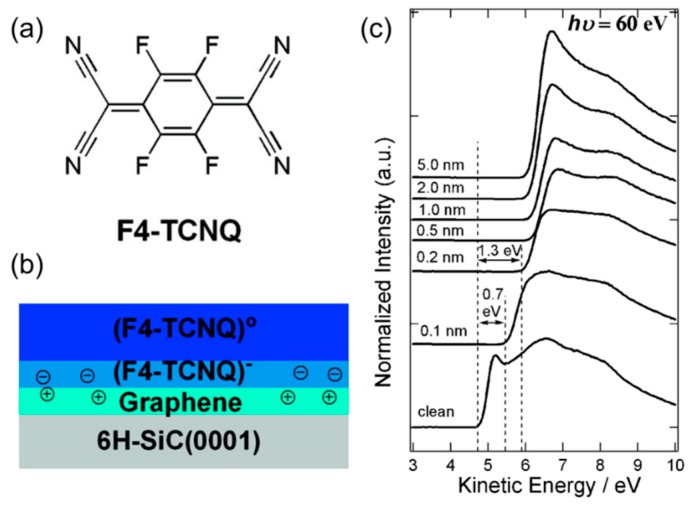
(**a**) Chemical structure of tetrafluoro-tetracyanoquinodimethane (F4-TCNQ) used as an electron acceptor for graphene; (**b**) Concept of charge transfer between graphene and the contacting F4-TCNQ layer; (**c**) Photoemission spectra recorded during the deposition of F4-TCNQ (low kinetic energy part). Reproduced with permission from [34]. American Chemical Society, 2007.

A study conducted by Chen et al. nicely illustrates the doping of graphene by organic molecules [34]. Here, the authors synthesized epitaxial graphene on a 6H-SiC substrate and performed synchrotron photoemission spectroscopy while evaporating in-situ a molecular film of tetrafluoro-tetracyanoquinodimethane (F4-TCNQ) (Figure 4a). As shown in Figure 4b, F4-TCNQ underwent direct charge transfer with graphene, accepting electrons to become negatively ionized. This can be equivalently considered as p-doping and the addition of extra holes to graphene. The photoemission spectroscopy results showed that the work function shift quickly saturates around 1.3 eV with 0.2 nm thick dopants (Figure 4c), suggesting that the effect is restricted to the very thin interfacial region of the dopant organic film. It was also found that the complete charge transfer is a consequence of the strong electron accepting character of F4-TCNQ. A weaker acceptor molecule C_60_ did not result in surface transfer doping on graphene.

**Figure 5:**
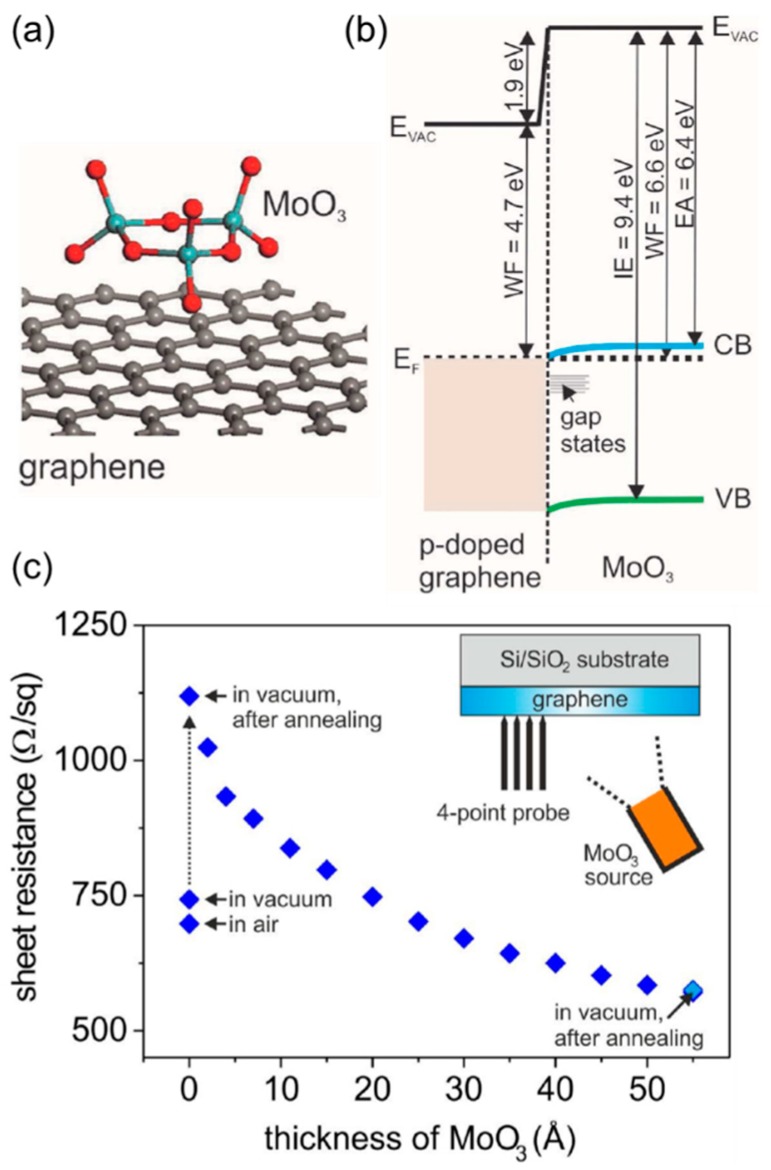
(**a**) Schematic illustration of MoO_3_ deposited on graphene surface; (**b**) Energy level alignment at graphene/MoO_3_ interface; (**c**) The evolution of sheet resistance in monolayer graphene. The overlapped final data points mean that the doped graphene is thermally stable (annealed at 140 °C). Reproduced with permission from [35]. Nature Publishing Group, 2014.

Metal oxides can be also an effective dopant for graphene. For instance, Meyer et al. reported a detailed investigation into the molybdenum trioxide (MoO_3_) thermally evaporated on chemical vapor deposition (CVD) grown and transferred graphene (Figure 5a) [35]. They observed a strong interface dipole (vacuum level shift of 1.9 eV) and substantial surface charge transfer that leads to the accumulation of electrons at the interfacial region of the MoO_3_ film and the equivalent p-type doping in graphene (Figure 5b). As shown in Figure 5c, the effect of oxide doping also manifested itself as a dramatic decrease in the sheet resistance of monolayer graphene. In few-layer graphene, the sheet resistance even decreased below 50 Ω/sq. Benefiting from both the efficient hole injection and excellent conductivity of doped graphene electrodes, the authors finally demonstrated organic light-emitting diodes (OLEDs) whose performance exceeds that of devices made with a conventional indium tin oxide (ITO) anode.

Additionally, there are two important notes to be made on graphene doping. Firstly, graphene can be ‘unintentionally’ doped. The substrate can have a polar nature which can slightly dope graphene even without any intentionally deposited dopants. Graphene is also readily oxidized in the ambient air to become apparently p-doped. This effect is similar to oxygen doping of organic semiconductors [36,37], and this tendency explains the deviation of the minimum conductance point in many graphene FETs from zero *V_G_*. An effective encapsulation can minimize further air-induced doping and shift of transfer curves [38]. On the other hand, Giovannetti et al. theoretically verified the charge-transfer doping of graphene by metal contacts, which can be regarded as another common source of unintentional doping in working devices [39]. Secondly, electrostatic doping can also play a role, and this needs to be taken into account along with (intentional or unintentional) chemical material doping. Because of the vanishingly small density of states near the Dirac point (Figure 1b), field-induced accumulated charges not only increase the conductivity but also significantly modulate the Fermi level of graphene. In other words, graphene’s work function is tunable by an electric field [40]. A chemically doped graphene (using charge-transfer molecules or oxides) will have a certain zero-field position of Fermi level solely determined by this doping, but when the material is put into an electric field, the Fermi level will change around this initial position.

In term of doping, one of the most extensively studied systems is FeCl_3_ doped graphene, first proposed in 2012, which can lead to an extremely low surface resistance of 8.8 Ω/sq based on an intercalation mechanism [41,42]. Later, it was confirmed that FeCl_3_-intercalated graphene exhibits an outstanding thermal and humidity stability [43], as well as a high work function of 5.1 eV, which is promising for ITO replacement [44]. The technological applications of this material have diversified, as evidenced by the recent demonstration of extraordinary linear dynamic range photodetectors [45], novel position-sensitive photodetector technologies [46], and ultra-bright large-area flexible lighting devices [47].

## 3. Hybridization Strategies

Nanostructured graphene materials can be used as an effective active component in a wide range of optoelectronic devices. In this section, advanced understanding will be established on how to build hybrid nanostructures which are useful in practical devices. Hybrid film structures will be first introduced, which will be followed by explanations on notable electrical and optical effects that are expectable from hybridizations. These effects are macroscopic descriptors, which have to be considered in relation to the material aspects (e.g., doping, size effects) reviewed in the previous section, to get a complete picture of device engineering.

### 3.1. Film Structures

**Figure 6:**
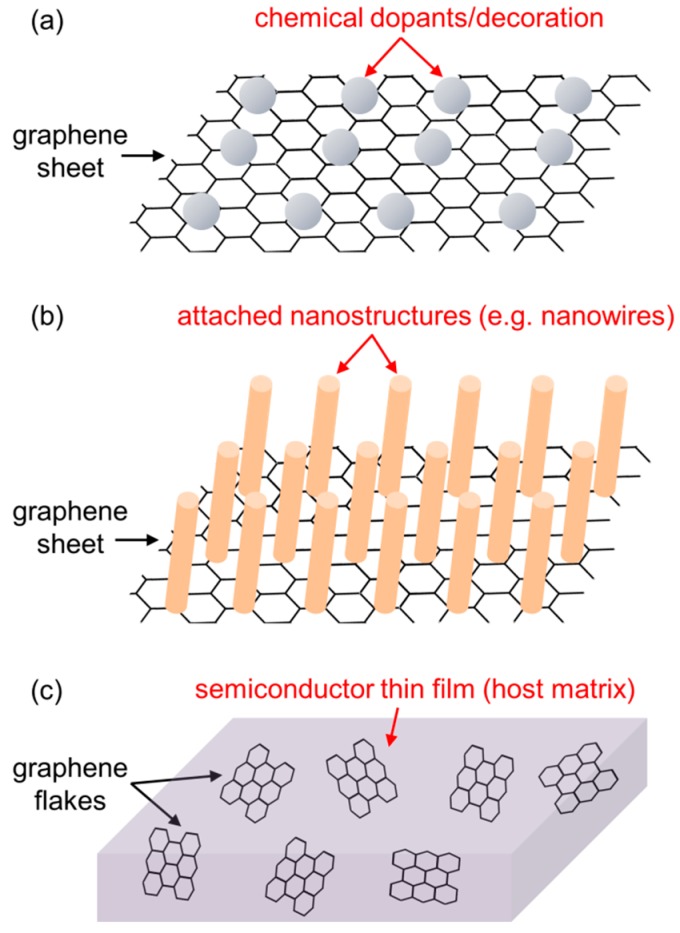
Three representative hybrid film structures that can be employed for a graphene-based active layer in optoelectronic devices. (**a**) Chemically decorated graphene; (**b**) graphene/nanostructure hybrid; (**c**) multicomponent blend.

In the literature, there are a huge variety of material compositions used in functional thin-film devices that can be regarded as a hybrid between graphene and other classical or emerging semiconductors. Here, an attempt is made to broadly classify them. Three structures are often encountered, and they are illustrated in Figure 6. First of all, a common factor in them is that they all try to develop a new function arising from direct interactions between graphene and its surroundings. Some of these structures use a large graphene sheet as an active layer and substrate at the same time, on which foreign species such as dopants and nanostructures can be attached or grown. Some other structures use a blended film as a composite-based active layer. It is important to note that there has been no comparable approach to symmetrically categorizing various graphene-based nanostructured channel materials. Nonetheless, part of the motifs in Figure 6 have been covered in several reviews [12,13,14]. More recent examples of each type of these hybrid structures will be discussed in detail in Section 4.

The first structure drawn as Figure 6a represents chemically decorated graphene systems. Organic molecules, nanoparticles (NPs), or other charge-transfer components are attached either covalently or based on weak van der Waals interactions, and these entities generally occupy only a small part of a graphene sheet that are selectively functionalized to accommodate them. From a functional point of view, these decorative materials mainly alter graphene’s chemical and electronic states such as oxidization levels, local band gaps, and conduction carrier types.

The second structure Figure 6b represents graphene/nanostructure hybrid systems. Nanotubes or nanowires directly grown on a graphene sheet can readily make up such a structure. The attached nanomaterials can eventually modulate graphene’s electronic properties via surface charge transfer, but they are mainly introduced for structural purposes; for instance, for maximizing interface areas.

The last structure, in Figure 6c, illustrates multicomponent blend films that include dispersed graphene nanoribbons or flakes. A wide variety of complex nanostructures can be produced by changing the types of bulk components, their mixing ratios, and film deposition methods. In this case, graphene can be considered as a kind of functional additive to a host medium, especially when it occupies only a small portion of the volume of the entire film.

Finally, it is worth mentioning that there are also possibilities for synthesizing hierarchically engineered materials that combine several motifs in Figure 5. One example can be a macroscopically blended film that features chemically decorated or nanostructure-anchored graphene nanoflakes.

### 3.2. Electrical Effects

**Figure 7:**
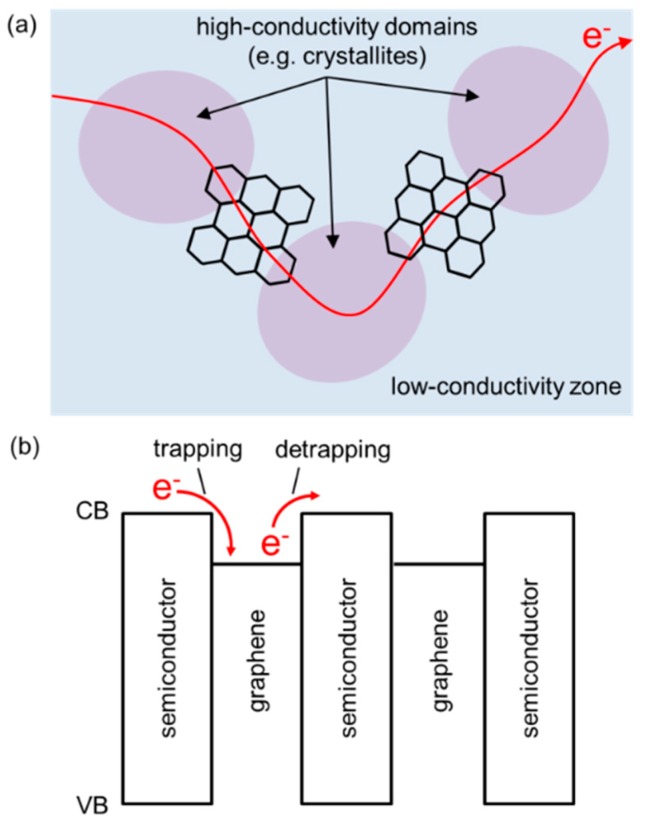
(**a**) Illustration for the preferred electronic transport pathways formed by inter-domain graphene bridges; (**b**) Energy diagram showing the trapping and detrapping of electrons that can be utilized for charge memory devices (CB: conduction band, VB: valence band).

When graphene is added to a semiconducting material, it can enhance the transport properties of that semiconductor. This is mainly because of the intrinsically high charge-carrier mobility (in excess of 10,000 cm^2^ V^−1^ s^−1^) [48,49], orders of magnitude larger than that of many general amorphous or polycrystalline thin-film semiconductor materials [50]. This effect can be understood by adopting the concept of a ‘conductive bridge’, as illustrated in Figure 7a. Even single-component channels can in fact consist of highly conductive domains that are surrounded and inter-connected by less conductive zones (e.g., voids, amorphous regions, grain boundaries). In this case, the overall charge transport is limited by inter-domain transport, and graphene blended into a film can effectively solve this problem by forming highly conductive inter-domain pathways. Previously reported statistical transistors where metallic islands passivate disconnected crystals or FETs with a carbon-nanotube/polymer hybrid channel showed similar benefits from enhanced connectivity or percolation [51,52]. However, it is important to note that, especially for transistor applications, care has to be taken not to make the entire channel too metallic (i.e., on-off ratio compromised). This would require careful optimization of nanoscale phase separation.

Another technologically relevant outcome of graphene incorporation is charge-based electrical memory effects. Semiconductor memories can be constructed by inserting charge-trapping components (e.g., metallic nanostructures) as an external floating gate or as an embedded carrier immobilizer [53,54,55,56]. For instance, Figure 7b illustrates the energetic situation in a semiconductor-graphene channel. In this architecture, graphene’s Fermi level can be tuned so that it can effectively trap charge carriers flowing through the semiconductor (programming), and these trapped carriers can be detrapped either naturally or by an electric field to recover the initial state (erasing).

### 3.3. Optical Effects

**Figure 8:**
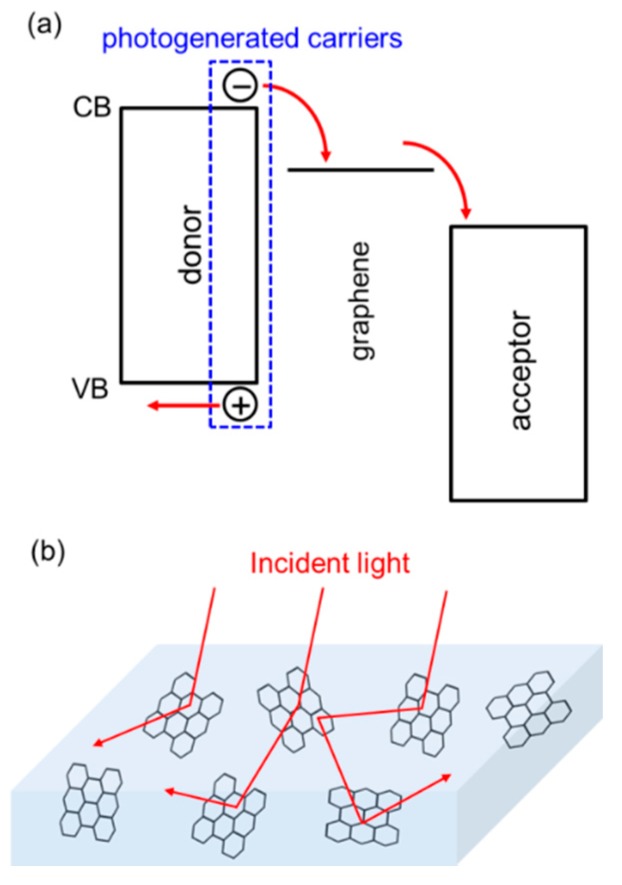
(**a**) Energy diagram showing the separation of photogenerated carriers aided by graphene (CB: conduction band, VB: valence band); (**b**) Illustration for the light scattering effect in a semiconductor-graphene hybrid film.

Technologically important optical devices such as light-emitting diodes (LEDs) or photovoltaics (PVs) are produced by stacking a multitude of layers that are designed to effectively perform the conversion between electricity and light. In addition to its use as an electrode, graphene can be used in the active layer(s) to provide an additional necessary energy level that helps to better carry out this conversion process. Figure 8a, for instance, shows critical energy levels involved in the operation of PVs. Assuming the light absorption mostly takes place in the donor material, an electron-hole pair (or exciton, depending on the material system), is generated at this material. Graphene with a tailored energy level can be introduced as an energetic bridge between the donor and acceptor materials, thus improving the charge separation and collection.

Another optical effect to note is light absorption enhancement, which is particularly useful for PVs or optical sensors. Distributed nanosized graphene flakes embedded in a semiconductor can basically function as a light scattering agent, similarly to traditionally used metal NPs [57,58]. While some of the scattered light can be eventually reflected back out of a device and become wasted, a carefully optimized scattering structure can yield an increase in absorption by elongating the optical path lengths (Figure 8b). Potentially, plasmonic near field effects can also contribute to the locally enhanced absorption and improved device performances [59,60,61].

## 4. Advances in Nanostructured Devices

Exploiting nanoscale material interactions and hierarchical synergies, a variety of optoelectronic devices have been demonstrated with hybrid graphene nanostructures as an active channel. Here, selected devices in the literature are reviewed, especially those that were published within the last 6–7 years. These real examples provide compelling evidence that graphene can be smartly engineered into diverse materials and structures to enhance their existing performances or to create novel functionalities. Thanks to graphene’s processing versatility, it has been successfully coupled with both organic and inorganic materials. Hybrid systems with these two classes of materials will be described, and both two-terminal (diode) and three-terminal (transistors) devices will be presented.

### 4.1. Organic-Based Systems

**Figure 9:**
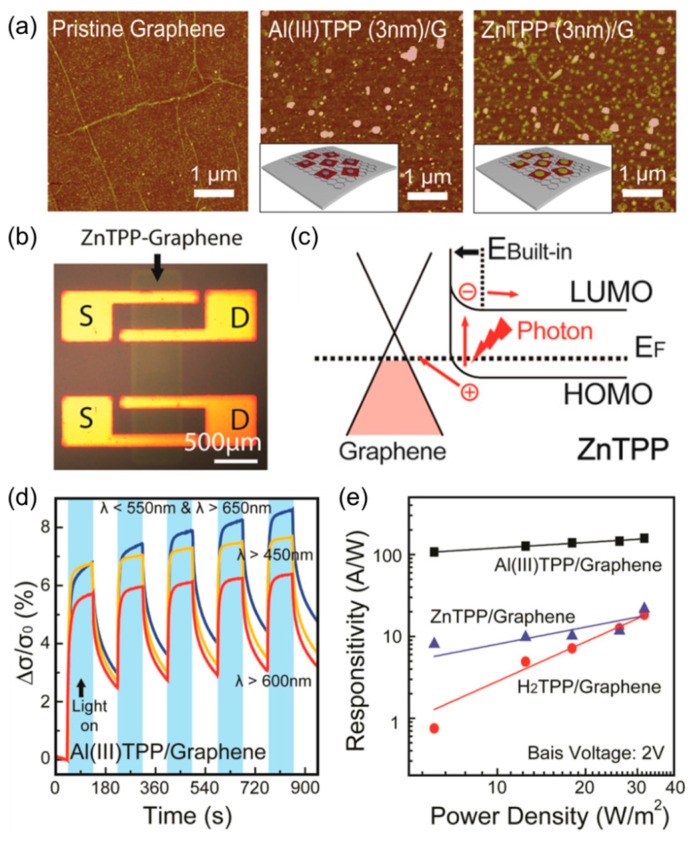
(**a**) AFM images of pristine, aluminum (III) tetraphenyl-porphyrin (Al(III)TPP), and zinc tetraphenyl-porphyrin (ZnTPP) functionalized graphene; (**b**) Optical image of a ZnTPP-graphene photodetector (S: source, D: drain); (**c**) Energy diagram for the device operation; (**d**) Change of relative photoconductivity upon exposure to light with different wavelengths at a *V_DS_* of 50 mV; (**e**) Responsivity as a function of light power density. Reproduced with permission from [62]. Institute of Physics, 2016.

Kim et al. proposed tunable organic functionalization of graphene for hybrid photodetectors (Figure 9) [62]. Two metalloporphyrins molecules, aluminum (III) tetraphenyl-porphyrin (Al(III)TPP) and zinc tetraphenyl-porphyrin (ZnTPP), were deposited on graphene sheets by vapor-phase metalation to form island structures (Figure 9a).

The lateral photodetector devices were fabricated using Au electrodes, as shown in Figure 9b. By observing the shifts of the charge-neutrality point of different graphene sheets, photo-induced doping was found to be a major mechanism for photodetection. Non-metallized H_2_TPP-graphene showed n-doping characteristics, while both Al(III)TPP- and ZnTPP-graphenes exhibited p-type doping (Figure 9c). The authors then carried out real-time measurements of photocurrents by using filtered light sources with different wavelengths (Figure 9d). At an optical power density of 31.7 W/m^2^ and a source-drain bias (*V_D_*) of 50 mV, H_2_TPP-, ZnTPP-, and Al(III)TPP-graphene exhibited a responsivity of 0.22 A/W, 0.54 A/W, and 5.36 A/W, respectively, proving substantial enhancement compared to pristine graphene and beneficial effects of metallization. This performance metric showed apparent voltage and power dependence, reaching a high value over 100 A/W in the case of Al(III)TPP-graphene at 2 V (Figure 9e). In this study, TPP-based organic molecules can be viewed as a sensitizer for graphene, providing substantial light absorption and photocarrier generation. Graphene’s good electrical conductivity should be another key contributor. It can be therefore summarized that the combination of these two properties has led to the impressive photoresponsivity of hybrid devices.

**Figure 10:**
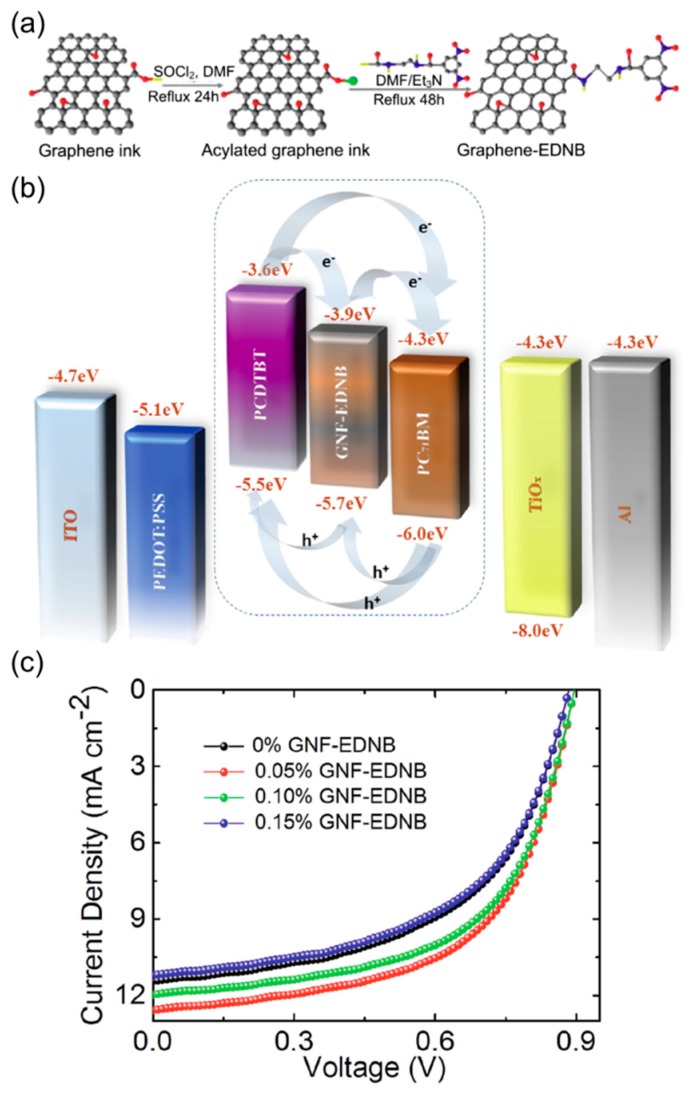
(**a**) Synthesis of 3,5-dinitrobenzoyl (EDNB)-functionalized graphene nanoflakes (GNF); (**b**) Energy diagram of a ternary PV illustrating the cascade effect; (**c**) Current density-voltage curves measured from devices with different GNF-EDNB concentrations under AM 1.5 condition. Reproduced with permission from [65]. Wiley-VCH, 2015.

Organic PVs have recently gained growing attention as a renewable energy technology [63]. Because of the low exciton diffusion length in organics, a donor-acceptor blend film is widely used instead of a planar p-n junction, thus forming a so-called bulk heterojunction [64]. Further extending this concept of ‘binary’ blend solar cells, Bonaccorso et al. proposed ‘ternary’ organic solar cells that include a functionalized graphene intermixed with a conventional donor-acceptor blend (Figure 10) [65]. Based on density functional theory (DFT), these authors first computationally evaluated the effect of 3,5-dinitrobenzoyl (EDNB) incorporation onto graphene to form graphene nanoflake (GNF)-EDNB (Figure 10a). By modulating the key parameters, e.g., anchoring site, number of epoxidic groups, and presence of a solvent, the energies of a number of materials were calculated. It was shown that all considered materials have a sizable bandgap near and over 2 eV, and that the position of their frontier orbitals were adjustable. The synthesized GNF-EDNB was solution-processable as an ink, thus enabling the solution-based co-deposition with an organic donor and an acceptor. Importantly, the composition of a ternary blend was chosen so that the GNF-EDNB can function as an energetic cascade between the two materials, providing an intermediate energy level that promotes exciton dissociation and carrier transport (Figure 10b).

As shown in Figure 10c, the PV cells with poly[*N*-9′-heptadecanyl-2,7-carbazole-*alt*-5,5-(4′,7′-di-2-thienyl-2′,1′,3′-benzothiadiazole)] (PCDTBT, donor) and [6,6]-phenyl-C71-butyric-acid-methylester (PC_71_BM, acceptor) were improved by a small amount of GNF-EDNB. With the optimum mixing ratio, an 18% increase in power-conversion efficiency (PCE) was obtained, from a PCE of 5.44% in binary cells (no graphene) to a PCE of 6.41% in ternary cells. This study clearly shows that the synthetic approach to fine-tuning of graphene flakes and the solution-based deposition is highly promising for simple production of efficient phase-controlled energy devices.

**Figure 11:**
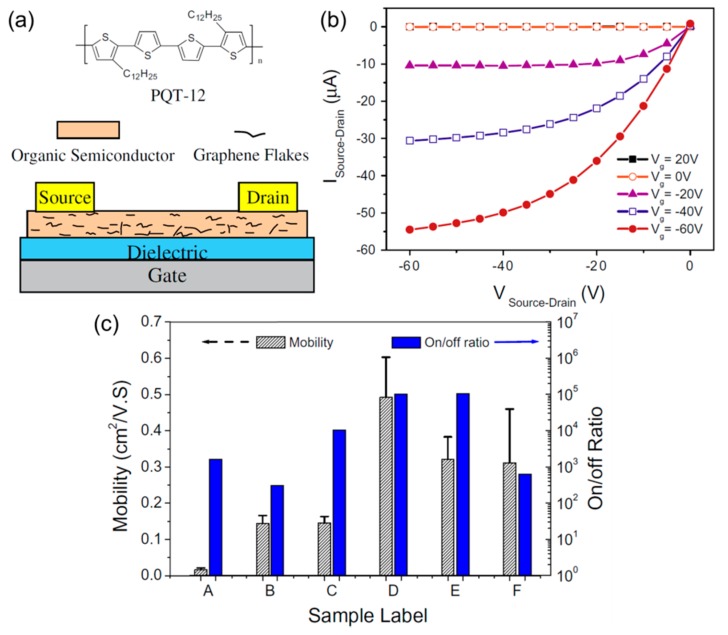
(**a**) Chemical structure of poly(3,3-didodecylquaterthiophene) (PQT-12) and the device structure of a hybrid FET; (**b**) Output characteristics of an optimized PQT-12/graphene transistor; (**c**) Mobility and on-off ratio for the samples with different fabrication conditions. Reproduced with permission from [66]. Elsevier, 2011.

In the case of FETs, Huang et al. proposed a method for enhancing the mobility of polymer transistors by graphene (Figure 11) [66]. The authors emphasized that controlled incorporation of graphene flakes into an organic channel can greatly enhance the charge-carrier transport without affecting the organic’s intrinsically low off-state conduction. Figure 11a shows the semiconductor material poly(3,3-didodecylquaterthiophene) (PQT-12) and the FET structure. A co-solution was prepared as polymer NPs and graphene flakes dispersed in ortho-dichlorobenzene and was spin-cast on a SiO_2_/Si substrate. As shown in Figure 11b, the inclusion of graphene did not seriously degrade the normal field-effect behavior, as evidenced by the good pinch-off and low leakage. At the same time, graphene provided favorable transport pathways in a polymer channel layer. A number of devices with different conditions, e.g., surface treatment, annealing, and graphene concentration, were tested (Figure 11c). The fully optimized FETs showed a hole mobility up to 0.6 cm^2^ V^−1^ s^−1^ and an on-off ratio of 10^5^. Therefore, this study shows that a hybrid graphene-organic channel is a promising platform for high-performance FETs, potentially useful for large-area flexible electronics and circuits.

**Figure 12:**
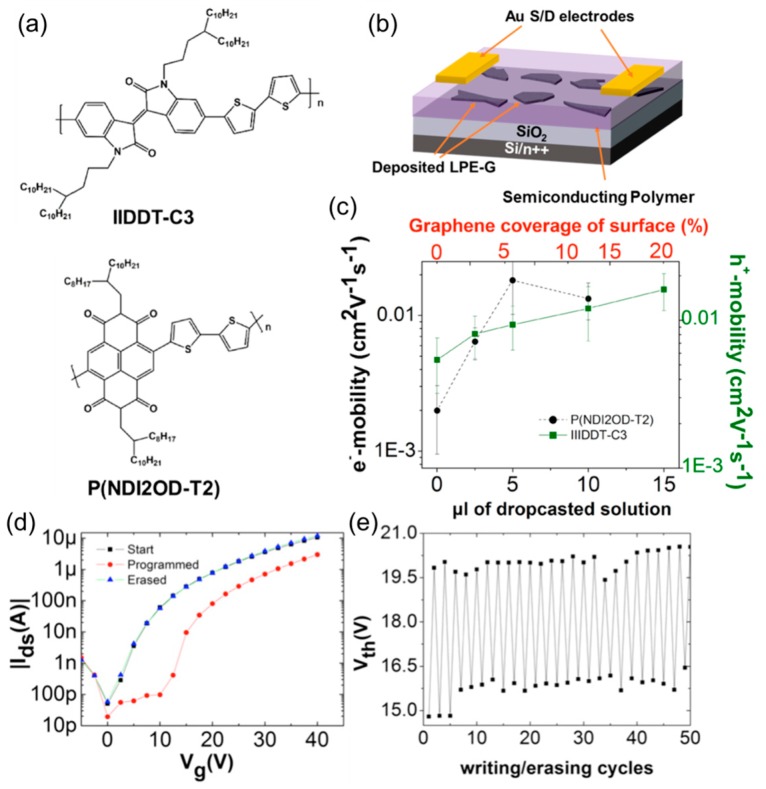
(**a**) Chemical structure of semiconducting polymers; (**b**) FET structure; (**c**) Charge-carrier mobility as a function of volume of the graphene solution and corresponding surface coverage; (**d**) Transfer curves for a P(NDI2OD-T2) device measured before and after a programming/erasing cycle (*V_D_* = 40 V); (**e**) Durable memory operation of a P(NDI2OD-T2) device shown as reproducible *V*_th_ shifts. Reproduced with permission from [67]. American Chemical Society, 2015.

Mosciatti et al. put forward a different strategy for making graphene-polymer hybrid FETs (Figure 12) [67]. In contrast to the co-deposition used for the devices in Figure 11, these authors separately and sequentially solution-deposited graphene and semiconductor. Both p-type poly[1,1′-*bis*(4-decyltetradecyl)-6-methyl-6′-(5′-methyl-[2,2′-bithiophen]-5-yl)-[3,3′-biindolinylidene]-2,2′-dione) (IIDDT-C3) and n-type poly[*N*,*N*′-9-*bis*(2-octyldodecyl)-naphthalene-1,4,5,8-*bis*(dicarboximide)-2,6-diyl]-*alt*-5,5′-(2,2′ bithiophene)] (P(DNI2OD-T2)) were used as a semiconducting material (Figure 12a). Liquid-phase exfoliated graphene (LPE-G) was drop-cast and thermally annealed at 415 °C either in air or in a nitrogen atmosphere. It was possible to systematically modulate the coverage of graphene on SiO_2_ (up to 50%), by changing the volume of a drop-cast solution up to 20 μL. The polymer semiconductor was then spin-cast to passivate LPE-G islands (Figure 12b). For both polymers, a transition from semiconducting to metallic channel was observed upon increasing the graphene contents. At roughly up to 10–15% surface coverage, the graphene ideally increased the field effect mobility while preserving an appreciable on-off ratio (Figure 12c).

Interestingly, the ionization energy of LPE-G was dramatically tunable by changing the thermal annealing duration and atmosphere. The graphene’s energy level could be placed either within or at the outside of the highest occupied molecular orbital (HOMO)–lowest unoccupied molecular orbital (LUMO) gap of the semiconducting polymers, and tunable transport regimes were observed in a hybridized channel. By adjusting the energy level of graphene, the FETs were addressed as a memory device. As shown in Figure 12d, a large threshold voltage (*V*_th_) shift was observed by applying programming and erasing gate pulses. Also, this electrical cycle was highly reproducible, as evidenced by the durability test results in Figure 12e. Therefore, this study shows that high-performance memory devices, which are an important building block for integrated circuits and sensor systems, can be fabricated by forming a single energy-matched hybrid channel, without needing to additionally deposit external floating gates and/or dielectrics.

The nanostructured graphene/organic systems introduced in this section can be compared to planar-junction devices where a sheet-type graphene forms a continuous interface with an organic film or crystal. Our previous review described many of these structures [14]. A recent study by Jones et al. illustrated an application of the continuous interface between graphene and rubrene single crystals, which allowed for the realization of high-sensitivity phototransistors based on efficient charge transfer [68].

### 4.2. Inorganic-Based Systems

**Figure 13:**
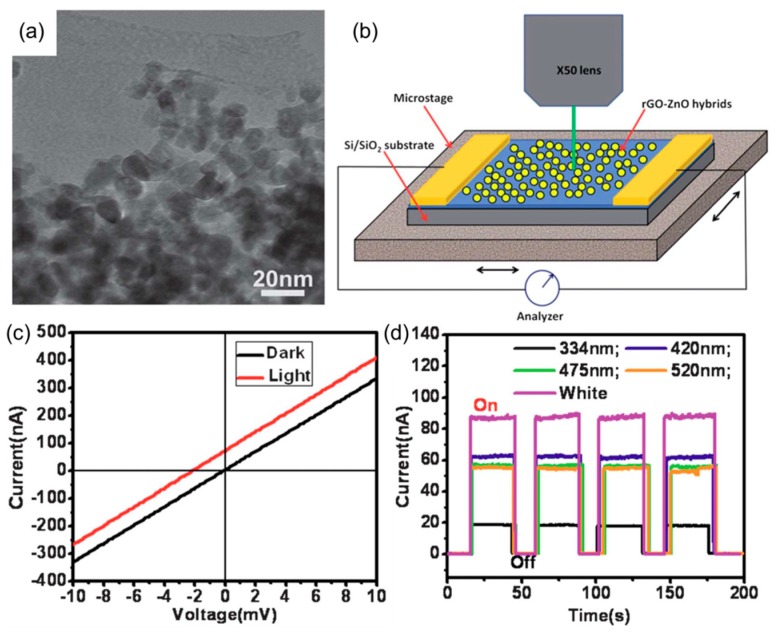
(**a**) Transmission-electron microscope (TEM) image of hybrid graphene-ZnO NPs; (**b**) Structure of a lateral diode photodetector and the set up for spatially resolved excitation and electrical measurement; (**c**) Current-voltage characteristics measured in the dark and under illumination; (**d**) Photocurrent responses measured at different wavelengths. Reproduced with permission from [71]. Royal Society of Chemistry, 2012.

Due to their strongly ionic character, metal-oxide semiconductors have a large band gap and are generally transparent to visible light [69,70]. Zhan et al. proposed a reduced graphene oxide (rGO)-ZnO hybrid nanostructure for efficient visible light photodetectors (Figure 13) [71]. This material was synthesized by a solvothermal method using GO as a template on which ZnO were grown as NPs (Figure 13a). The hybrid rGO-ZnO channel was incorporated into a lateral diode type device, which was characterized by photocurrent measurements. In addition to large-area surface illumination, a more sophisticated focused laser excitation system was used (Figure 13b), and this proved that the photoconductivity is not contact-region dominated (as in many graphene-base detectors) but is a direct consequence of charge transfer between the two materials throughout the bulk of a film. Interestingly, the devices showed a high responsivity to the visible light. Figure 13c is a response to the white light illumination, and it interestingly showed both photocurrent and photovoltage, implying the possibility of self-powered operation in a PV mode. As shown in Figure 13d, the devices were also highly sensitive to the monochromatic lights over visible wavelengths. Detailed structural and chemical analyses provided the reason for the visible light detection. A high temperature annealing (700 °C) used for thermal reduction of GO not only recovered a non-oxidized state of graphene, but it also resulted in carbon doping of ZnO. Because of the doping, new electronic states within the ZnO bandgap were created, into which low-energy photons can excite electrons which are then transferred to graphene to contribute to the electrical currents. In short, this study demonstrated the power of combined structural and chemical interaction between graphene and an inorganic nanostructure that drives the optoelectronic performance.

**Figure 14:**
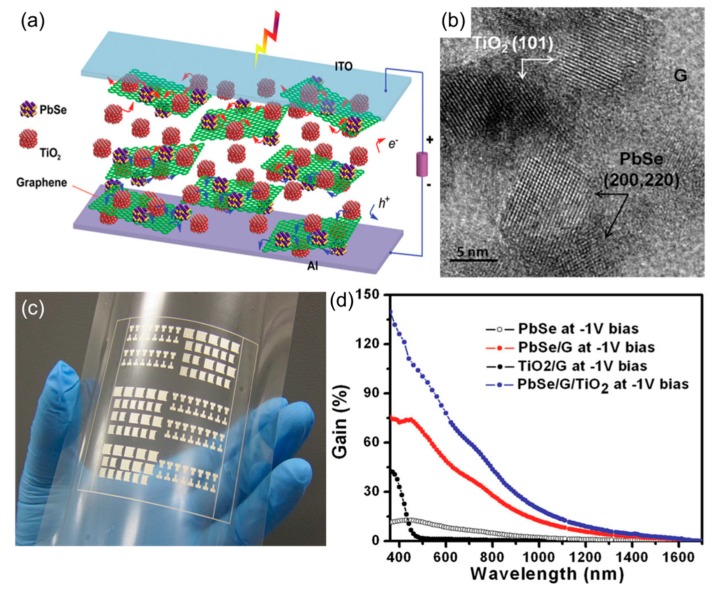
(**a**) Illustration of the working mechanism of a PbSe-TiO_2_-graphene photodetector; (**b**) TEM image showing the inorganic crystals on graphene; (**c**) Large-area printed photodetector arrays on plastic; (**d**) Photoconductive gain as a function of excitation wavelength in different compositions. Reproduced with permission from [72]. Wiley-VCH, 2012.

As another example of hybrid diodes, Manga et al. demonstrated vertical photodetectors based on a ternary PbSe-TiO_2_-graphene active layer (Figure 14) [72]. In this material system, graphene first served as a growth template for PbSe and TiO_2_ nanoscrystals, allowing for the formation of tightly intermixed nanocomposites (Figure 14a,b). Also, graphene was shown to be able to effectively extract holes from the excited PbSe quantum dots and electrons from TiO_2_, providing an ambipolar pathways for charge separation and collection. Furthermore, the hybrid material was grown in solution and was entirely solution processable at a low temperature (annealed at 160 °C). This allowed for the fabrication of photodetectors on plastic (Figure 14c). Another important achievement was broad-band detection. PbSe, with a small bandgap (ca. 0.9 eV), mostly absorbed visible to infrared (IR) photons, while a large band gap of TiO_2_ (ca. 2.7 eV) makes it sensitive to the ultraviolet (UV) region. As shown in Figure 14d, the three-component system combined the specific sensitivities of these two photoactive materials with a high photoconductive gain, as graphene promoted free carrier generation from both materials. Therefore, this study provided convincing proof of the dual function of nanostructured graphene, i.e., as a nanomaterial growth mediator and charge-carrier transporter, which is useful for the realization of flexible high-performance optical devices.

**Figure 15:**
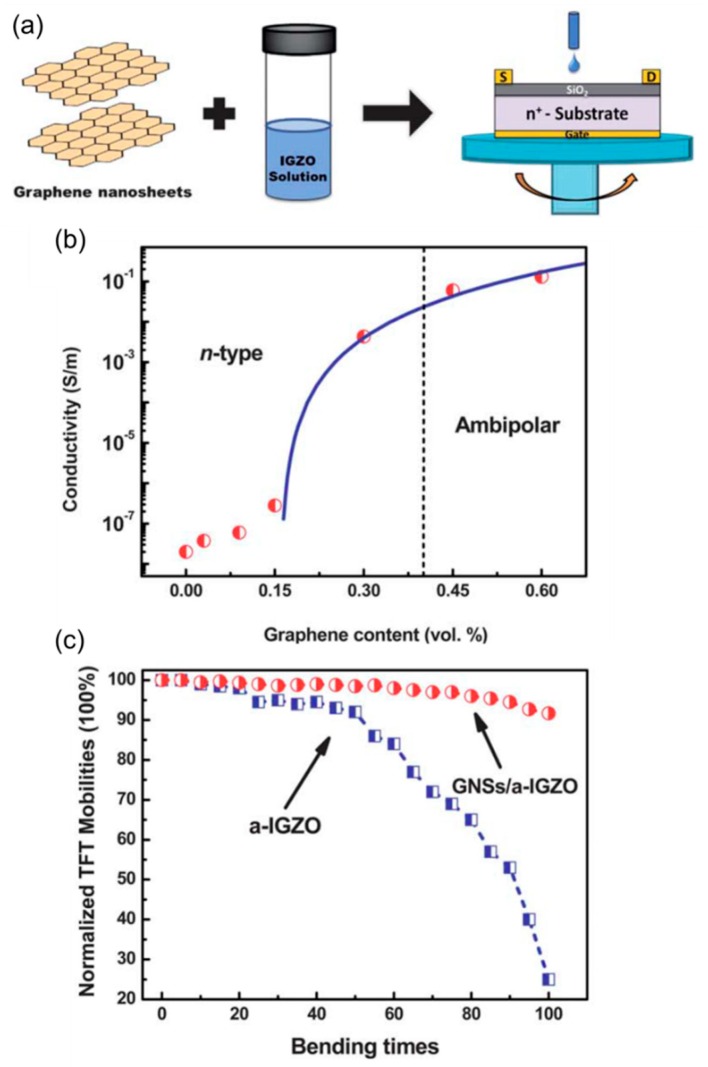
(**a**) Fabrication process for the FETs with a soluble graphene–In-Ga-Zn-O (IGZO) hybrid; (**b**) Electrical conductivity of the blend film as a function of volume percentage of graphene content. Symbols are experimental data and the solid line is a fit to the percolation theory; (**c**) Mechanical stability against bending for the pristine IGZO and graphene-hybridized FETs. Reproduced with permission from [76]. Royal Society of Chemistry, 2013.

Solution-processable In-Ga-Zn-O compounds (IGZO) are promising n-type semiconductors for printed large-area electronics [73,74,75]. Dai et al. demonstrated transparent IGZO-graphene mixed channel based high-performance FETs (Figure 15) [76]. In this work, exfoliated graphene nanosheets (GNSs) were added to a sol-gel amorphous IGZO (a-IGZO) solution, and the mixed solution was one-step deposited on a transistor substrate by spin coating (Figure 15a). By changing the volume fraction of GNSs in the a-IGZO matrix (from 0.03 to 0.6 vol %), different transport mechanisms were identified (Figure 15b). Before the percolation threshold (<0.15 vol %), the GNSs were disconnected while effectively making conductive bridges. This led to a dramatic increase in the effective electron mobility, from 0.82 cm^2^ V^−1^ s^−1^ in an IGZO-only device to 23.8 cm^2^ V^−1^ s^−1^ in a GNSs/a-IGZO FET. At higher concentrations, off-state currents started to increase as the percolation takes place. Eventually, at graphene contents over 0.45 vol %, balanced ambipolar behavior was observed, reflecting the dominant transport through well-connected GNSs. After finishing this mechanism analysis on rigid FETs on Si, the authors also presented low-voltage flexible devices using an ultra-thin glass substrate and a high dielectric constant Ta_2_O_5_ insulator. Interestingly, the formation of a hybrid channel also enhanced bending stability. The hybrid FET showed only 8% reduction in mobility after 100 times of bending, while the pristine device suffered from serious 70–80% decrease (Figure 15c). This study, therefore, was an important development for inorganic-based high-performance FETs; the mobility enhancement by graphene was successfully extended to an amorphous semiconductor, and the discovered mechanical stabilization effect was well elucidated.

**Figure 16:**
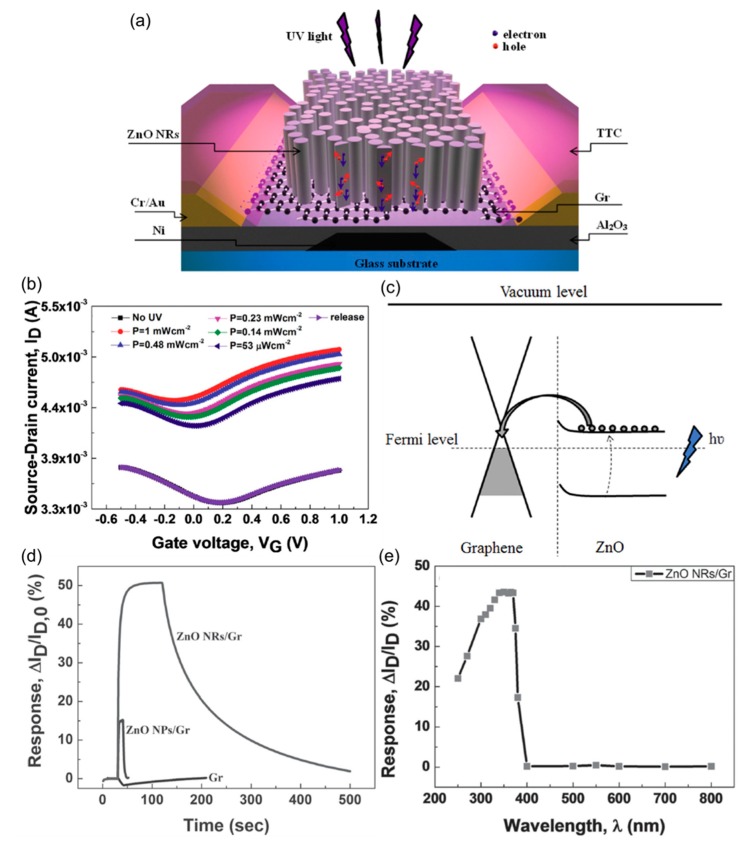
(**a**) Structure of a graphene-ZnO nanorod (NR) hybrid FET; (**b**) Transfer curves of a graphene-ZnO NR device under different UV irradiation powers (*V_D_* = 1 V); (**c**) Energy diagram illustrating the sensing mechanism; (**d**) Response of graphene (Gr), ZnO NPs/Gr, and ZnO NRs/Gr FETs at the UV intensity of 2 mW/cm^2^; (**e**) Wavelength-dependent response of a ZnO NRs/Gr device. *V_G_* = 0 V and *V_D_* = 1 V for (**d**,**e**). Reproduced with permission from [77]. Wiley-VCH, 2015.

Dang et al. recently published research on photosensitive hybrid FETs based on a graphene sheet channel decorated with ZnO nanorods (NRs) (Figure 16) [77]. These authors attached either ZnO NPs or NRs on a pre-deposited graphene channel by solution-phase casting and a hydrothermal method (Figure 16a) and compared the properties of these two hybridized materials to pristine graphene (Gr). A focus was placed on the UV sensing applications. As shown in Figure 16b, a substantial increase in *I_D_* was observed for the ZnO NRs/Gr device. The current increases as the incident power increases, and this photocurrent effect was reversible. A large band gap of ZnO allowed for the efficient absorption of UV photons, and the electron transfer from ZnO to graphene enabled electrical read out. The real-time UV response shown in Figure 16d revealed a striking difference between the pristine and the functionalized channels.

UV irradiation on pristine device resulted in a decrease in *I_D_*, and this was explained by the desorption of molecular p-type dopants on the graphene surface. The ZnO NPs-based channel exhibited a lower photoresponse than the NR counterpart, and this was attributed to the smaller absorption cross-section and the larger bandgap due to the quantum confinement. Because ZnO acted solely as the absorber medium with a defined bandgap, a high selectivity of photodetection (maximum response at wavelengths around 365 nm) was obtained. Interestingly, this is in sharp contrast to the system reviewed in Figure 13, where ZnO was chemically doped and the mid-gap states contributed to the visible light absorption. Therefore, this study indicates that it is possible to allocate distinct functions to each component in a hybrid system, in this case an optical function to ZnO and an electrical function to graphene; yet, their interfaces still dominate the overall integrated behavior of devices.

## 5. Future Perspectives

The overview given in the previous section is convincing evidence that nanostructured graphene hybrid optoelectronics has witnessed a considerable growth over a relatively short period of time. Many new deposition methods, advanced composites, device designs, and fabrication techniques have been developed, and they have all significantly contributed to the current status of the field. However, important issues exist that need to be tackled in the near future for this new research area to get necessary momentum and turn into a viable technology.

Firstly, material processing can be further improved. For instance, strongly acidic or toxic chemical environments are still widely utilized for growth, synthesis, and structural control, and this is not desirable in terms of sustainability and commercialization. Aqueous routes or other green chemistry approaches have been appreciated in organic electronics, where harsh chemicals and hazardous solvents have been traditionally used [78]. This can provide a benchmark for environmentally friendly and widely applicable protocols for graphene-hybrid processing. Also, the method for reducing the process temperature deserves further investigation. Hybrid materials with GO or sol-gel semiconductor precursors have required a relatively high-temperature post-treatment for the purpose of chemical reduction, activation, purification, or cross-linking. Recent reports have shown that photochemical reaction and/or combustive synthesis can help reduce the activation temperature, while additional opportunities also arise, for instance, for the enhancement in doping efficiencies and stability [79,80,81]. Advanced low-temperature processing based on such recent developments is expected to raise the applicability of hybrids for plastic or paper-based optoelectronics.

Secondly, better structural control can be an added value. The hybrid system, by its nature, often develops very complex nanostructures with distinct phase separation mechanisms. Failing to fully manipulate the distribution of multiple components in a composite-based channel, for instance, may lead to limited reproducibility and large-area uniformity. In addition to the control over the blend solution and annealing, area-selective deposition through anchoring groups and/or the addition of functionally inactive scaffolds can help improve the controllability over phase separation or guide the formation of new nanostructures [73,82].

Finally, new hybrid materials and devices are of great interest. This review has provided a clear indication that many creative and original systems are being tested. Given the improved understanding of graphene handling and the emergence of new semiconductors, there should be other potentially important hybrid architecture that is worth looking at. As another benchmark, the traditional boundary between organic and inorganic material systems has been challenged by many recent developments, such as 4G thin-film solar cells [61] or polymer-doped metal-oxide semiconductors [83]. This means that new hybrid structures can incorporate mixed organic-inorganic (semi)conductors with nanographene serving as a multi-functional mediator, and other advanced systems based on new growth, mixing, functionalization, and patterning techniques can be envisaged. Although not extensively dealt with in the present article, light-emitting device platforms might be another important and promising direction for graphene optoelectronics. It is worth referring to the realization of high-performance OLEDs using a work-function gradient in graphene anodes [84] and the demonstration of graphene-channel ultrafast broad-spectral-range light emitters [85]. Such recent developments reveal the potential of new photonic devices that may employ functionalized graphene as both contacts and emitters with highly adjustable properties.

## 6. Conclusions

Nanostructured and modified graphene possesses a great utility for enabling new optoelectronic devices with hybridized structures and properties. Combining the functional diversity with the processing versatility, including solution printing, novel device concepts have been demonstrated with a variety of semiconductors and nanomaterials. In many representative systems, graphene has been used as a sheet-type channel that is decorated with additives, or it has been mixed into a host that serves as a bulk semiconducting medium and dispersion agent. Various devices such as planar photodetectors, multi-component PVs, hybrid FETs, transport-tunable memories, quantum-dot sensitized diodes, and nanowire UV detectors have been suggested with organic and inorganic matching components. Here, graphene successfully served a range of roles including chemical hosts/dopant materials, photocarrier acceptors, energetic cascades, percolation bridges, charge floating gates, growth templates/linkers, ambipolar transporters, electrical transducers, and so on. This array of novel devices has shown that graphene-based hybrid architecture can be a major building block for next-generation optoelectronics. Major issues regarding processing, controllability, efficiency, sustainability, scalability, and performance need to be solved in the near future, and the technological developments will be further accelerated, benefiting from extensive research efforts that will be continuously put into this highly promising area.
